# Dengue outbreaks: unpredictable incidence time series

**DOI:** 10.1017/S0950268819000311

**Published:** 2019-03-01

**Authors:** A.F.B. Gabriel, A.P. Alencar, S.G.E.K. Miraglia

**Affiliations:** 1 Instituto de Ciências Ambientais, Químicas e Farmacêuticas (ICAQF), Laboratório de Economia, Saúde e Poluição Ambiental, Universidade Federal de São Paulo – UNIFESP, São Paulo, Brazil; 2 Instituto de Matemática e Estatística, Universidade de São Paulo – USP, São Paulo, Brazil

**Keywords:** Forecast, dengue, SARIMA

## Abstract

Dengue fever is a disease with increasing incidence, now occurring in some regions which were not previously affected. Ribeirão Preto and São Paulo, municipalities in São Paulo state, Brazil, have been highlighted due to the high dengue incidences especially after 2009 and 2013. Therefore, the current study aims to analyse the temporal behaviour of dengue cases in the both municipalities and forecast the number of disease cases in the out-of-sample period, using time series models, especially SARIMA model. We fitted SARIMA models, which satisfactorily meet the dengue incidence data collected in the municipalities of Ribeirão Preto and São Paulo. However, the out-of-sample forecast confidence intervals are very wide and this fact is usually omitted in several papers. Despite the high variability, health services can use these models in order to anticipate disease scenarios, however, one should interpret with prudence since the magnitude of the epidemic may be underestimated.

## Introduction

Dengue fever presents high levels of infection being reported in many tropical and subtropical localities populated by *Aedes aegypti* mosquitoes, the main vector of the disease [1].

In the 21st century, Brazil has become the country with the highest number of reported cases of dengue in the world, reaching the first place in the international ranking for total cases of the disease [2]. In this country, the southeastern region has been very affected, especially São Paulo state, with high numbers of dengue cases being reported. In 2015 exclusively, more than 745 600 cases were reported in São Paulo state, representing 1732 cases per 100 000 inhabitants. Two municipalities in this state, Ribeirão Preto and São Paulo, have been highlighted due to the high incidence of dengue [3–6]. In the period from 2000 to 2015, the annual incidence rate in the municipality of Ribeirão Preto ranged from 9 to 4903 cases per 100 000 inhabitants. In the municipality of São Paulo, the annual incidence rate ranged from 1 to 837 cases per 100 000 inhabitants, considering the same period.

In the face of this scenario, obtaining detailed information on when and where dengue outbreaks occurred in the past can be a useful guide to predict the magnitude and severity of future epidemics and thus allow adequate allocation of resources to better public health interventions [7]. Therefore, time series analysis tools have been used to predict the occurrence of infectious diseases such as dengue [7–9], malaria [10] and influenza [11]. Hence, the current study aims to analyse the temporal behaviour of dengue cases in the municipalities of Ribeirão Preto and São Paulo, in order to forecast the monthly number of dengue cases in 2016.

## Methodology

### Study area

Ribeirão Preto is a municipality in São Paulo state, Brazil, with a south latitude of 21°10′ and a longitude of 47°50′ west. It occupies an area of about 651 km^2^, with 127 km^2^ being in urban perimeter [12, 13]. The Brazilian Institute of Geography and Statistics (or IBGE) estimated its population as 674 405 inhabitants [14] and its economy is based on agribusiness, mainly the sugar-alcohol sector and citriculture.

São Paulo is a Brazilian municipality, capital of São Paulo state, with a south latitude of 23°32′ and longitude of 46°38′ west. It is the most populous city in Brazil with more than 12 million inhabitants and is the main financial, mercantile and corporate centre of South America [15–17].

### Data collection

The number of dengue cases (monthly basis) and the population information of the municipality of Ribeirão Preto were obtained through the database of the City Hall of Ribeirão Preto [18, 19]. For the municipality of São Paulo, the number of dengue cases were obtained through the DATASUS database and the City Hall of the city of São Paulo [20–22].

### Statistical analysis

In order to analyse the monthly number of dengue cases in each city until 2015, seasonal autoregressive integrated moving average (SARIMA) time series model was proposed since the SARIMA takes into account the seasonality, possible nonstationarity and all autocorrelations. To satisfy all the assumptions of the usual SARIMA model, as homoscedastic uncorrelated errors with Gaussian distribution and to include outliers in epidemic periods, the model was fitted to the logarithm of the number of cases plus 1 [5, 8], summing 1 to the number of cases to avoid zero counts.

The SARIMA model [23] was chosen after fitting several models with different SARIMA(*p*, *d*, *q*)(*P*, *D*, *Q*) specifications, where *d* and *D* correspond respectively to the number of usual differences and seasonal differences necessary to achieve stationarity, *P* and *p* are the autoregressive orders and *Q* and *q* are the moving average orders. A first candidate model is the one which minimises the Akaike information criteria (AIC) [24], corresponding to the one that maximises the likelihood, penalising an increase in the number of parameters, avoiding overfitting.

After fitting the model with the best AIC using the maximum likelihood method, a residual analysis is performed to check the validity of all assumptions of the model. The final model must have all valid assumptions. The residual analysis consists of the time series plot of observed and predicted monthly number of cases; a time series plot of residuals, the residual autocorrelation function, the Ljung–Box tests [25] and the residual qq-plot to check the normality.

For the final model, the significance of all parameters was tested using the Wald test and non-significant terms were removed from the model. After choosing the final model, the monthly number of dengue cases in 2016 was forecasted with their respective 95% confidence intervals. All tests considered the 5% level of significance and all the analyses were executed in R software.

Federal University of São Paulo Ethical Committee approved this study under process number 3696290616.

## Results

**Fig. 1.**
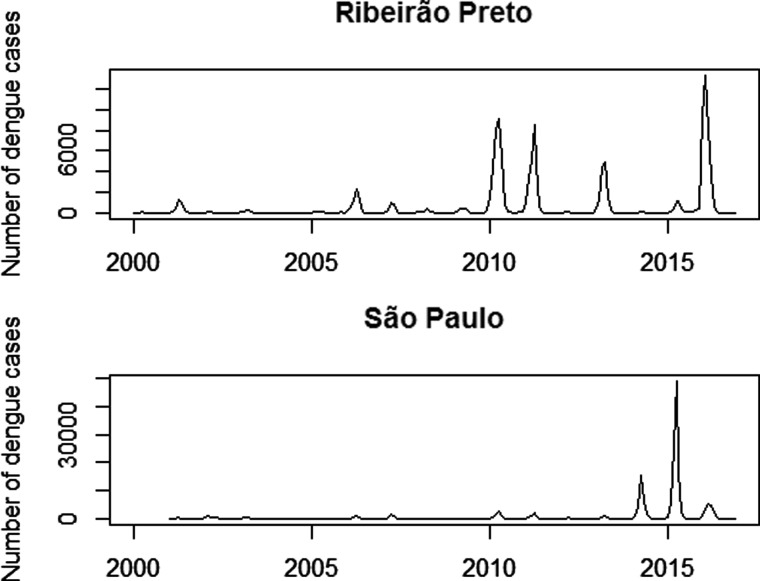
Monthly number of dengue cases: in the municipality of Ribeirão Preto from 2000 to 2016 and in the municipality of São Paulo from 2001 to 2016.

In the period from 2000 to 2016, more than 11 million cases of dengue were reported in Brazil, of which 2 080 584 were in the state of São Paulo. The municipalities of Ribeirão Preto and São Paulo were responsible for more than 14% of the total cases in this state. The number of monthly dengue cases in both cities is shown in Figure 1, in order to analyse behaviour over time.

It is verified that the number of dengue cases shows a cyclical behaviour, especially in Ribeirão Preto. It should be observed that in the years 2010, 2011, 2013 and 2016, Ribeirão Preto presented a large number of individuals with the disease, totalising about 101 thousand infected individuals, that is, 15 945 cases per 100 000 inhabitants. On the other hand, the years 2000, 2002, 2004, 2012 and 2014 presented a low incidence with few reported cases. In São Paulo, the years 2014 and 2015 were the ones with the highest number of dengue cases, with more than 129 440 cases, representing 1119 cases per 100 000 inhabitants. The years 2004 and 2005 were the years with the lowest incidence of dengue. In addition, it is observed that the appearance of cases is increasing in the first months of each year, coinciding with the seasons of the summer, that is, the incidence of dengue presents an annual seasonal cycle.

Thus, considering the seasonality of the disease, it was possible to adjust SARIMA models with different indications of the components *p*, *d*, *q*, *P*, *D*, *Q*, whose SARIMA(0,1,0)(2,0,0)_12_ presented the lowest AIC for both Ribeirão Preto and São Paulo. The residuals obtained fitting this model present significant autocorrelations until the lag 6, indicating that SARIMA(6,1,0)(2,0,0)_12_ is more appropriate and in fact it satisfied all the model assumptions. The next step was to estimate the parameters of the proposed model.

### Municipality Ribeirão Preto

Concerning the data from Ribeirão Preto, we observed that the AR1 and AR3 terms were not significant and were removed from the model. The results for the final fitted model are shown in Table 1.

**Table 1.** Estimates, standard error and *p*-value of SARIMA(6,1,0)(2,0,0)_12_ model – Ribeirão Preto

	Estimate	Standard error	*Z* Stat.	*p*
AR2	0.266	0.070	3.822	<0.001
AR4	−0.185	0.071	−2.590	0.010
AR5	−0.163	0.068	−2.385	0.017
AR6	−0.213	0.071	−2.999	0.003
SAR1	0.194	0.076	2.575	0.010
SAR2	0.284	0.076	3.723	<0.001

**Fig. 2.**
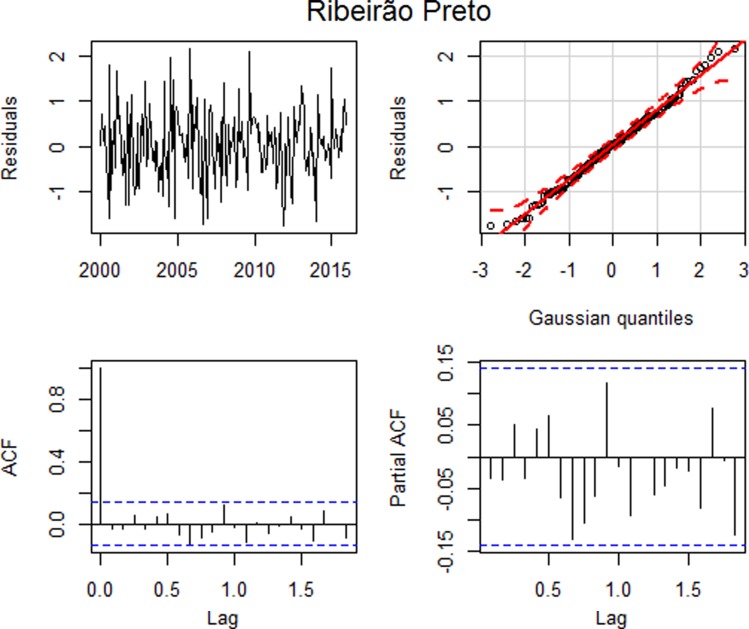
Residual plots: (a) time series plot, (b) qq plot, (c) autocorrelation and (d) partial autocorrelation – Ribeirão Preto.

After estimating the parameters of this model, we assessed their adequacy by analysing their residuals (Fig. 2).

Figure 2(a) suggests that the standardised residuals estimated from this model should behave as an independent and identically distributed sequence with a mean of zero and a constant variance. The qq plot, Figure 2(b), shows that the standardised residuals for the model approximated a normal distribution. Based on the Ljung–Box test, the hypothesis of all autocorrelations up to lag 15 are null (*p* = 0.3395), suggesting that the residuals behave as a white noise. This can be seen in Figures 2(c) and (d), where the graphs of the autocorrelation function and the partial autocorrelation function suggest that the autocorrelations are jointly non-significant, that is, the autocorrelations approach of zero. Thus, all assumptions were satisfied and the model error variance is 0.6914.

**Fig. 3.**
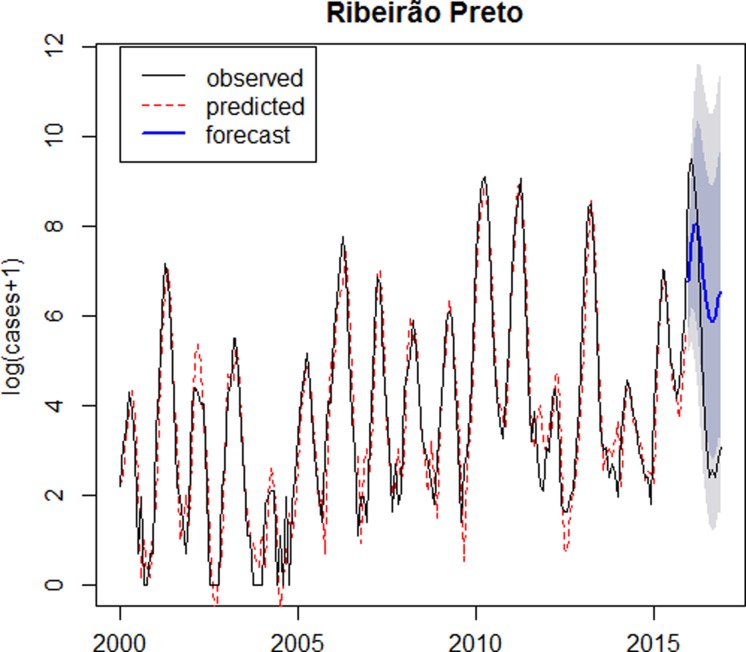
Logarithm of number of dengue cases observed plus 1 between 2000 and 2016 (solid line), logarithm of number of dengue cases predicted by the SARIMA(6,1,0)(2,0,0)_12_ model (dotted line), forecast for 2016 (solid line highlighted) and confidence intervals (shaded) – Ribeirão Preto.

As the model is satisfactory, it was possible to carry out the forecast for 2016, which is represented in Figure 3.

The SARIMA(6,1,0)(2,0,0)_12_ model closely fits dengue in Ribeirão Preto, however for the out-of-sample forecasts, the confidence intervals in the log scale are very wide; this shows that the forecasts are not very accurate.

### Municipality São Paulo

For the data from São Paulo, the first three autoregressive coefficients were not significant (*p* = 0.9024 – Wald), these terms were removed from model. The estimates of parameters for the final model are shown in Table 2.

**Table 2.** Estimates, standard error and *p*-value of SARIMA(6,1,0)(2,0,0)_12_ model – São Paulo

	Estimate	Standard error	*Z* Stat.	*p*
AR4	−0.210	0.071	−2.940	0.003
AR5	−0.189	0.073	−2.571	0.010
AR6	−0.187	0.072	−2.595	0.009
SAR1	0.458	0.074	6.227	<0.001
SAR2	0.252	0.077	3.258	0.001

**Fig. 4.**
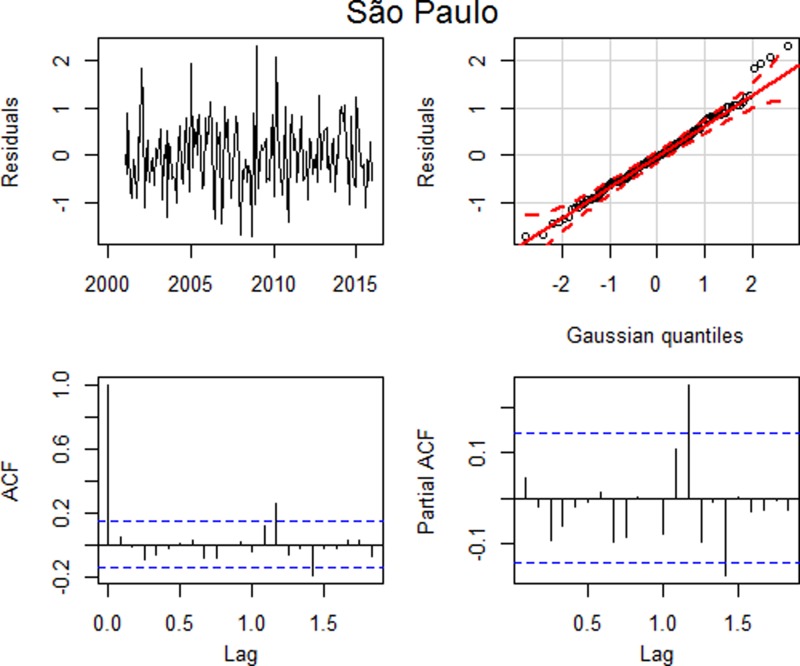
Residual plots: (a) time series plot, (b) qq plot, (c) autocorrelation and (d) partial autocorrelation – São Paulo.

After estimating the parameters of this model, we assessed their adequacy by analysing their residuals (Fig. 4).

Similarly to the analysis of the data performed for Ribeirão Preto, the residual analysis for São Paulo indicated that this model was adequate, with uncorrelated residuals up to lag 15 (*p* = 0.1481), despite a higher autocorrelation in lag 14, and with approximately normal distribution (Fig. 4) and the model error variance estimate is 0.4571.

**Fig. 5.**
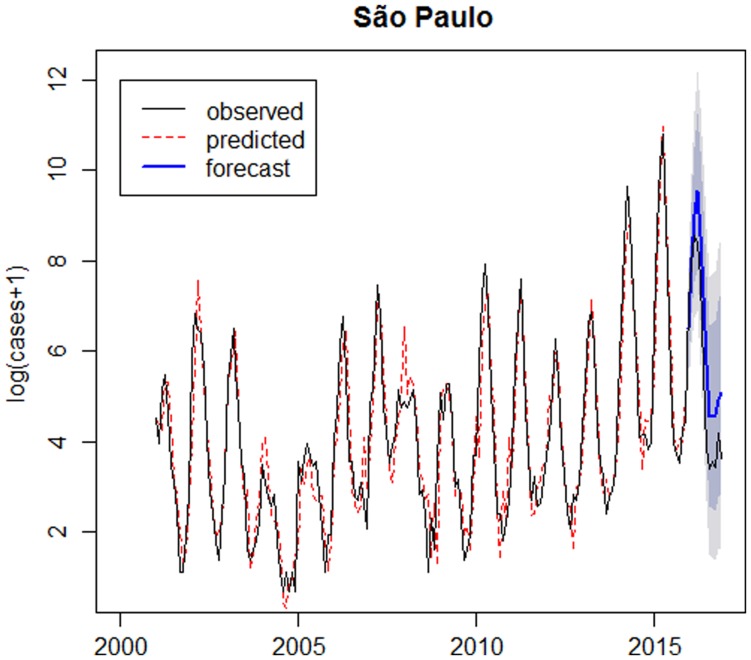
Logarithm of number of dengue cases observed plus 1 between 2001 and 2016 (solid line), logarithm of number of dengue cases predicted by the SARIMA(6,1,0)(2,0,0)_12_ model (dotted line), forecast for 2016 (solid line highlighted) and confidence intervals (shaded) – São Paulo.

As the model is appropriate, it was possible to carry out the forecast for 2016, which is represented in Figure 5.

### Evaluating the forecast number of cases

**Fig. 6.**
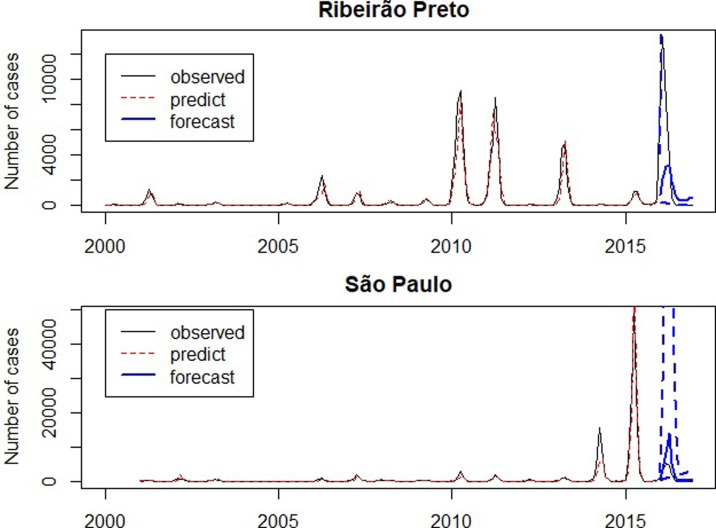
Number of dengue cases between 2001 and 2016 (solid line), predicted number of dengue cases by SARIMA models (dotted line), forecast for 2016 (solid line highlighted) and confidence intervals (dashed).

The graph presented in Figure 6 compares the observed, the predicted and forecast number of cases (not in the logarithm scale). In São Paulo, the predicted number of cases during the outbreak in 2014 was lower than the observed, since it was the first outbreak in São Paulo and the forecast for 2016 was larger than the observed. In Ribeirão Preto the predicted values are close to the observed number of cases and forecasts for 2016 are smaller than the observed number of cases.

**Fig. 7.**
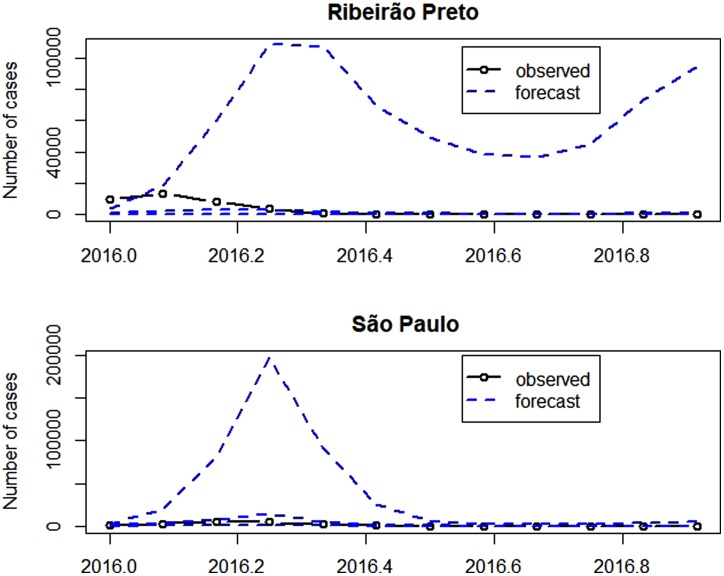
Number of dengue cases in 2016 (solid line with dots), forecast for 2016 (dashed) and confidence intervals (dashed).

Figure 7 presents the observed and the forecasts of the number of cases in 2016. The forecast 95% confidence intervals are so wide that their upper limits reach more than the double of the monthly number of cases ever observed, highlighting the large variability of forecasts. Exemplifying this situation, for illustrative purposes, the 95% confidence intervals for the number of cases in April 2016 were [66; 114 479] in Ribeirão Preto and [983; 197 178] in São Paulo. Also in April 2016, there were 3554 cases in Ribeirão Preto and 4524 cases in São Paulo. The observed number of cases belongs to the intervals, however, they are very wide.

## Discussion and conclusion

Looking ahead to future scenarios of the diseases distribution in the population and recognising the capable factors of interfering with this distribution allows decision making and planning to reduce the burden of diseases [26]. Thus, the time series analysis tools, in particular the SARIMA models, have been widely used by several authors (Table 3) to forecast the occurrence of outbreaks of infectious diseases, such as dengue. Additionally, for comparison purpose, our results are shown in Table 3.

**Table 3.** Studies using SARIMA models for estimation and prediction of time series

Reference	Area of study	Period	Data	Model
Luz *et al*. [8]	Rio de Janeiro, Brazil	1994–2004	Monthly	SARIMA(2,0,0)(1,0,0)_12_
Hu *et al*. [27]	Queensland, Australia	1993–2003	Monthly	SARIMA(1,0,0)(2,1,0)_12_
Gharbi *et al*. [9]	Guadeloupe, French West Indies	2000–2006	Weekly	SARIMA(0,1,1)(0,1,1)_52_
Martinez *et al*. [28]	Campinas, Brazil	1998–2008	Monthly	SARIMA(2,1,2)(1,1,1)_12_
Martinez & Silva [5]	Ribeirão Preto, Brazil	2000–2008	Monthly	SARIMA(2,1,3)(1,1,1)_12_
Bhatnagar *et al*. [7]	Rajasthan, India	2001–2010	Monthly	SARIMA(0,0,1)(0,1,1)_12_
Dela Cruz *et al*. [29]	Philippines	2005–2010	Monthly	SARIMA(1,0,1)(0,1,1)_12_
Phung *et al*. [30]	Can Tho, Vietnam	2003–2010	Monthly	SARIMA(1,1,1)(1,1,0)_12_
**Our results**	**Ribeirão Preto and São Paulo, Brazil**	**2000–2015** **2001–2015**	**Monthly**	**SARIMA(6,1,0)(2,0,0)**_**12**_ **SARIMA(6,1,0)(2,0,0)**_**12**_

In the current study, the analysis of time series allowed the development of SARIMA models, which satisfactorily fit the dengue incidence data collected in the municipalities of Ribeirão Preto and São Paulo, in addition to forecasting the number of dengue cases for a subsequent year. Our fitted model has a larger order of the autoregressive term because it was necessary to take into account the residual autocorrelation to meet all the model assumptions. This means that the choice of an appropriate SARIMA model depends on each particular analysed time series and for each case a complete residual analyses must be accomplished, after choosing a candidate model using the AIC criteria. In this study, for the municipality of Ribeirão Preto, from 2000 to 2015, the SARIMA model (6,1,0)(2,0,0)_12_ presented the best fit. On the other hand, Martinez & Silva [5] concluded that the SARIMA model (2,1,3)(1,1,1)_12_ was the one that had the best fit for the incidence of dengue cases in the period from 2000 to 2008, for the same municipality. In this sense, we can see that the different series require different SARIMA models.

The results presented in Table 3 showed that the predicted number of dengue cases in Ribeirão Preto depends on the number of cases in the previous 2 to 24 months. In addition, the monthly incidence of dengue observed in 2016 was significantly higher than the number predicted by the SARIMA model. In fact, out-of-sample forecasts follow the past observed behaviour and may not be credible to forecast the number of dengue cases in epidemic years, such as 2016, since the large number of reported cases may be a consequence of the lack of immunity of the population exposed by the first dengue virus, making the outbreak unpredictable [5]. Only in 2016, more than 35 000 cases of dengue fever were confirmed, being considered the largest epidemic in the city's history.

In São Paulo, the predicted number of dengue cases in a given month depends on the number of dengue cases occurring in the previous 24 months. The year 2015 presented the largest epidemic ever occurred in the city of São Paulo, with more than 100 400 confirmed cases. Thus, a decline in the number of dengue cases in the following year was expected due to the immunity acquired by the population exposed to one of the four viral serotypes of dengue [31]. However, a forecasting model assumes that a distribution pattern will be repeated in the future [32], so the forecast for 2016 followed the same trend as in 2015 and the expected number of cases for 2016 was higher than the observed number of cases.

Several papers displayed in Table 3 also presented out-of-sample forecasts, but, in general, they present confidence intervals for the forecasts only in the log-scale. Moreover, they omit the corresponding interval for number of cases, because they would be very wide. These wide intervals indicate that we must use these forecasts prudently.

Although dengue predictive models have difficulties in maintaining the accuracy of the prediction, due to their epidemiology is influenced by a combination of factors [32], it is essential to carry out similar research for an early identification of diseases. Elimination of dengue as a burden for public health can only be achieved through the integration of vector control and vaccines [33]. On the other hand, efforts to anticipate disease scenarios may prioritise a better combination of vector control interventions according to a magnitude of the epidemic, as well as helping to provide subsidies for structuring health care services.
